# Training effect on sex-based differences in components of the Shepard and Metzler mental rotation task

**DOI:** 10.1186/s40101-022-00314-z

**Published:** 2022-11-11

**Authors:** Tomoaki Kozaki

**Affiliations:** Fukuoka Women’s University, 1-1-1 Kasumigaoka, Higashi-ku, Fukuoka, Japan

**Keywords:** Mental rotation, Gender difference, Reaction time

## Abstract

**Background:**

Spatial ability has been reported to indicate sex-based differences in humans, mainly assessed by Shepard and Metzler mental rotation task (SM-MRT). Most performances in earlier studies have been evaluated by the mean value of reaction time and/or accuracy. The performance indexes might not be sensitive measures of mental rotation. Sex-based differences in the performance might also be involved in the spatial experience of the subject at the time. This study observed variations in components of the SM-MRT over repetition.

**Methods:**

Male (*n* = 17) and female (*n* = 17) subjects completed 20 days of repeating the SM-MRT. The slope and intercept of the function performance (reaction time) to the angular disparity are calculated; the slope of this function indexes the mental rotation (main-process), and the intercept indexes the other sub-processes.

**Results:**

A significant main effect of sex was obtained on the slope. The intercept also showed a tendency toward statistical difference. The interactions between the sexes and the day were not significant for the indices. Statistical testing for coefficient of variations (CV) indicated no sex-based difference in the effect of the intercept throughout the experiment day. The CV of the slope, however, showed tendencies toward sex-based difference from days 7 to 12.

**Conclusions:**

The difference between the sexes in performance on the slope was sustained throughout the experimental period. A few female subjects who demonstrated larger slope values than male subjects caused the sex difference. The learning rate of mental rotation may be an inherent spatial ability.

## Introduction

Some cognitive functions have been reported to indicate sex-based differences in humans. One of these is spatial ability, mainly assessed by the mental rotation task (MRT) [1–3]. The MRT devised by Shepard and Metzler (SM-MRT) [4] is a task in which subjects decide whether two figures are the same (one of them is a mirror-image). The two figures are rotated, and the angular differences are from 0° to 180°. Since the 1970s, some studies have shown that male subjects outperform female subjects in the SM-MRT [5, 6]. However, the same cannot be said for some other studies [7, 8].

Recent studies have measured the performance of various trial types of the SM-MRT. These were trials with mirror-image figures and with structural differences from one figure to the other [9]. They obtained significant differences in performance among the various types of MRT, suggesting that the performance of MRTs depends not only on mental rotation but also on other cognitive sub-processes (e.g., comparing the terminal arm directions of the figures or counting blocks of the figures). Most performances in earlier studies have been evaluated by the mean value of reaction time and/or accuracy. The performance indexes might not be sensitive measures of mental rotation because performing an MRT involves several distinct cognitive processes [10]. These are distinguished by the following sub-processes: (a) mental representation of an object, (b) rotation of the object, (c) comparison of the two objects shown, (d) determination of the identity of the two objects, and (e) motor response. Hooven et al. [11] proposed a method that isolated mental rotation ability (step “b” above) from other abilities involved in the task. Performance aspects, such as reaction time, often indicated a strong positive relationship with the degree of angular disparity between the two objects. From Hooven et al.’s [11] method, the slope and intercept of the function relating performance to the angular disparity are calculated; the slope of this function indexes the mental rotation, and the intercept indexes the other sub-processes. Our previous studies [6, 12] indicated no significant relationship between the slope and the intercept, meaning that these indexes measure different processes for solving the SM-MRT.

I have found significant differences between the sexes in performances on the slope, suggesting sex-based differences in mental rotation [6]. However, spatial experience (e.g., spatial games and majors in college) might improve the performance of the SM-MRT [13–16]. Some studies also attributed the improvement of the SM-MRT’s performance to repeating the task [12, 17, 18]. These findings imply that sex-based differences in the performance of the SM-MRT are involved in the spatial experience of the subject at the time. Most previous studies have obtained SM-MRT performances from a few trials. Therefore, the sex-based differences in mental rotation or another sub-process may become smaller or less after the subjects have enough spatial experience. This study observed variations in components of the SM-MRT over repetition. Furthermore, some neuro-imaging studies [7, 19] obtained sex-based differences in cerebral activation patterns during the SM-MRT, implying sex-based differences in strategies for solving the SM-MRT. I also examined strategy variation by correlations between the components of the SM-MRT. In the task of this study, mirror images, structurally similar to the figures, were employed because structural differences in the figures (e.g., the number of blocks of the figures) might involve performance [9].

## Methods

### Subjects

Thirty-four subjects participated in the study with written consent: 17 male subjects aged 21–26 years (mean + S.D.; 22.5 + 1.24) and 17 female subjects aged 20–25 years (22.2 + 1.29). There was no significant difference in ages between the sexes. I confirmed that all of them had had spatial experience (video games, sports, or art), since they were junior high-school students. They were recruited from the Department of Design, Kyushu University, Fukuoka, Japan. All subjects were right-handed as determined by a 13-item handedness questionnaire [20] and had normal color vision determined by the Ishihara test [21].

### Spatial ability assessments were based on the Shepard and Metzler mental rotation task

**Fig. 1:**
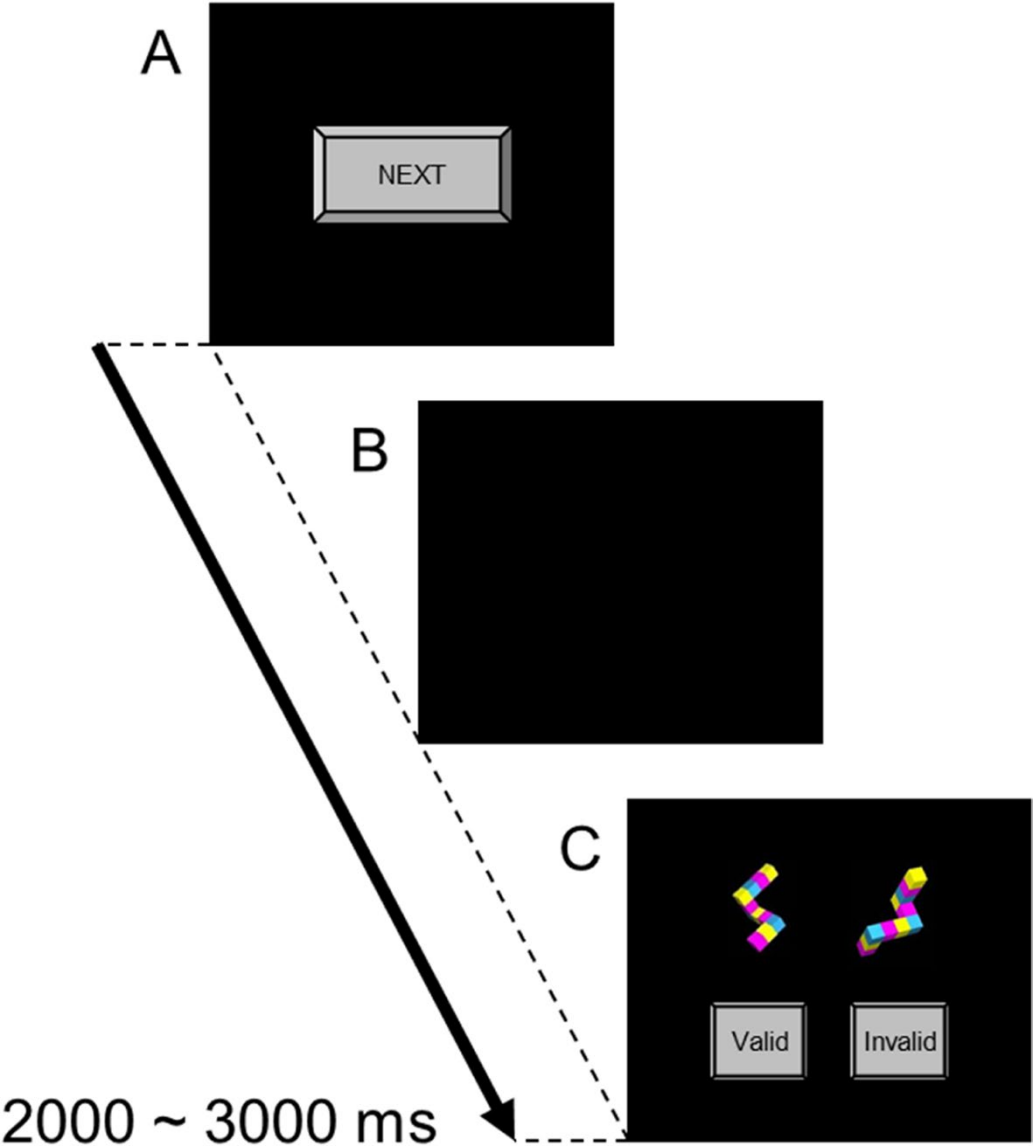
Three-dimensional mental rotation task. In each trial, the subject was required to press the “Next” click-on button (**A**), whereupon the button disappeared (**B**). Two figures were displayed 2000~3000 ms after the disappearance of the button (**C**)

Spatial ability was assessed by a 3-dimensional MRT (Fig. 1), a modified version of the SM-MRT [4]. Each trial began with the presentation of the “Next” click-on button on the monitor. Subjects were required to click the button using a computer-attached mouse (Fig. 1A), whereupon it disappeared (Fig. 1B). Two figures were simultaneously displayed on a computer monitor (Fig. 1C) 2000–3000 ms after the disappearance of the “Next” button. One of the two figures was rotated along a vertical axis, and the other figure was rotated in the plane of the drawings. The angular differences between the two figures varied from 0° to 180° at 20° rotation intervals. Subjects were asked to click the “Valid” button on the monitor using the mouse as soon as they determined that the two drawings portrayed object congruent with a three-dimensional shape and were tasked with clicking the “Invalid” button as soon as they determined that the two drawings depicted objects representing different three-dimensional shapes. The button-clicking response caused the three-dimensional objects to disappear and clocked the reaction time from the presentation of the figures to the response. The “Next” button was presented again 2000 ms after the response. The subjects performed 60 trials of the task; the stimuli represented identical objects in 40 trials (the valid trials) and an object and its mirror-image in the remaining 20 trials (the invalid trials). Four valid trials were conducted at each rotation angle. Trials of validity and angular differences were randomized for each task performed.

### Procedures

All subjects participated for 20 days. To minimize circadian rhythm influences, the experiment was performed in the afternoon (14:00–17:00 h). The interval between experiments was less than three days. The task was separated into two-block trials, with each block consisting of 30 trials. Subjects were allowed to have a break before the trials as each trial began with the subjects pressing the “Next” click-on button. To perform linear contrast on the reaction time with the rotation angles of each subject, they were instructed to respond as correctly as they could in each trial. If the number of trials with correct responses was less than 54 trials (the rate of correct response was less than 90%), the incorrect trial was repeated at the end of the task again. Therefore, we obtained three or four invalid trials correct with responses on each rotation angle.

### Data analysis

Reaction times (RTs) in error and outlier trials were omitted from data analysis: an outlier was defined as the time exceeding (the mean + 2 × S.D.) of two trials on each day. I performed linear regression on the reaction time scores with rotation angles of each subject, and then computed the slope (main-process; mental rotation) and intercept (sub-process; other than mental rotation) functions of the reaction time for each subject. The mean RTs (the composite of main- and sub-processes) of each subject were computed each day. The number of trials with incorrect responses was counted.

Sex-based differences in the data were analyzed using repeated measure two-way analysis of variance. ANOVAs were performed using SPSS (version 22.0; IBM). The coefficient of variations (CV) (%) and the coefficient of determination (CD) were computed on each data by Microsoft Excel (version 2019, Microsoft). Significant differences between CVs were tested using the R package cvequality [22]. Differences where *p* < 0.05 were considered statistically significant.

## Results

**Fig. 2:**
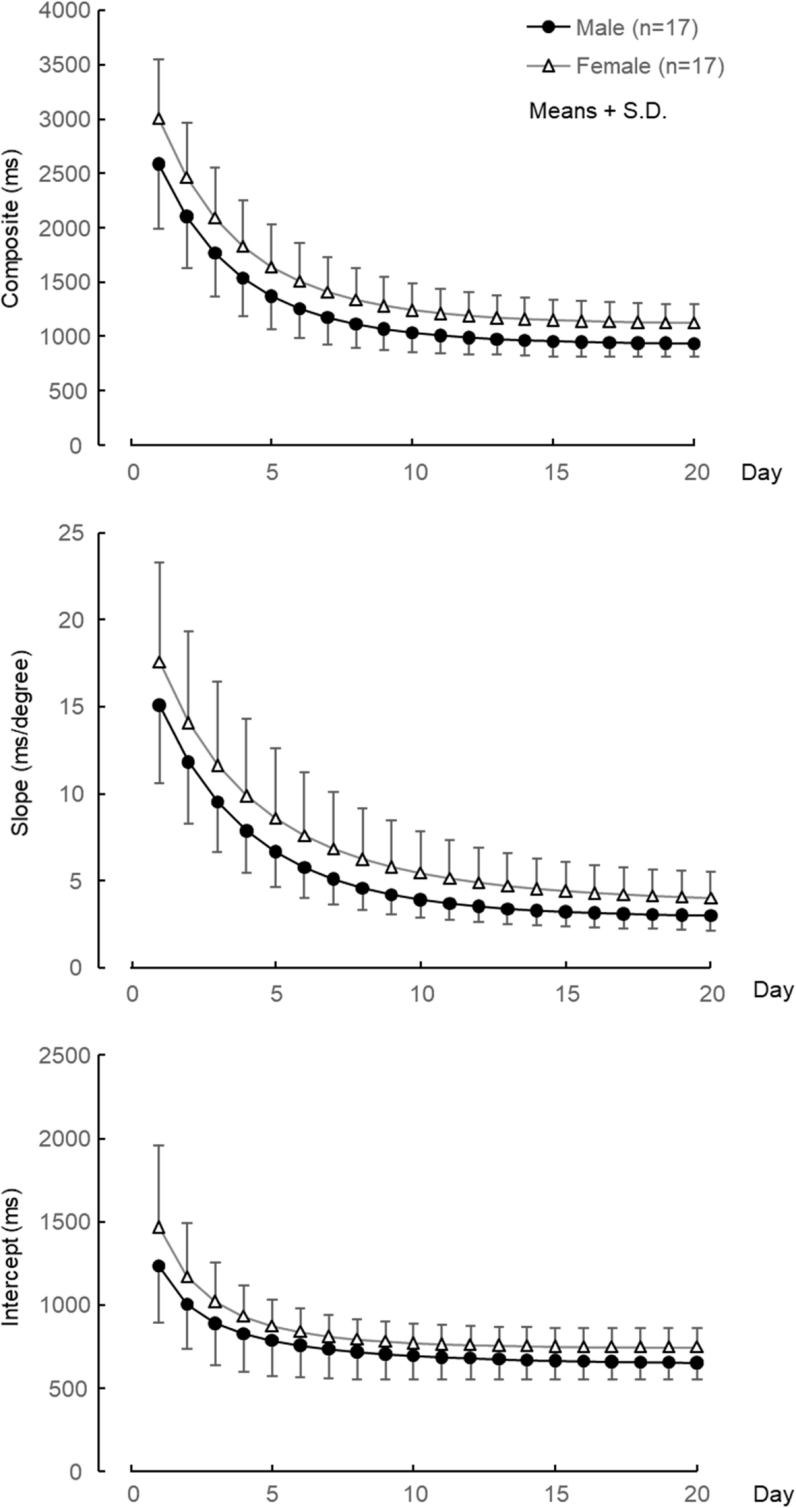
Mean values of components of MRT for 20 days. Composite of main- and sub-processes (upper), main-process “slope” (middle), and sub-process “intercept” (lower). Black circles are male and white triangles are female

Figure 2 shows the mean values of the composite, the slope, and the intercept on the male and female subjects. Significant main effects of the experiment day were found on all indices; the composite: *F*(19, 608) = 289.8, *p* < 0.001; the slope: *F*(19, 608) = 174.7, *p* < 0.001; and the intercept: *F*(19, 608) = 78.8, *p* < 0.001. A significant main effect of sex was obtained on the composite: *F*(1, 32) = 7.5, p = 0.01 and the slope: *F*(1, 32) = 4.4, *p* = 0.05. The intercept showed a tendency toward statistical difference: *F*(1, 32) = 3.6, *p* = 0.07. The interactions between the sexes and the day were also not significant for the indices: the composite *F*(19, 608) = 1.3, *p* = 0.15; the slope *F*(19, 608) = 0.7, *p* = 0.85; and the intercept *F*(19, 608) = 1.1, *p* = 0.38.

**Fig. 3:**
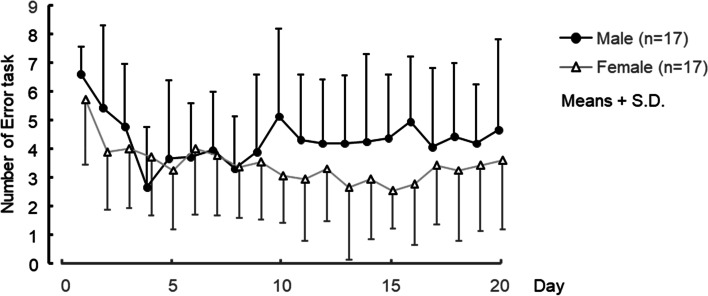
Mean values of the number of incorrect trials for 20 days. Black circles are male and white triangles are female

Although the interaction between the sexes and the experiment day was not significant on the number of trials with incorrect responses (Fig. 3) — *F*(19, 608) = 1.43, *p* = 0.11 — a significant main effect of experiment day was obtained: — *F*(19, 608) = 3.55, *p* < 0.001. A main effect of sex also tended to be statistical significant: *F*(1, 32) = 3.70, *p* = 0.06.

**Fig. 4:**
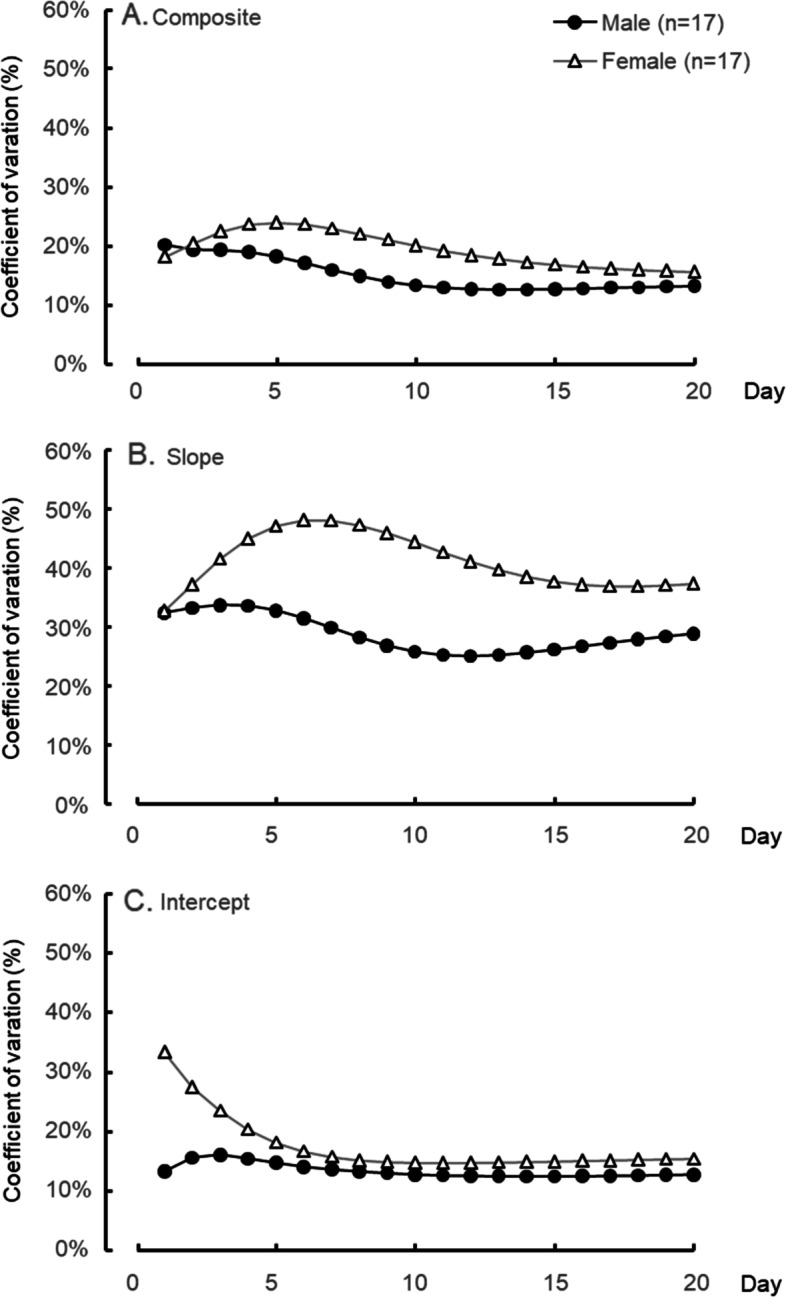
Coefficient of variation (CV) of the components for 20 days. Composite of main- and sub-processes (upper), main-process “slope” (middle), and sub-process “intercept” (lower). Black circles are male and white triangles are female

Figure 4 presents the CV (S.D./mean) for each index. Statistical testing for CV indicated no sex-based differences in the effects of the composite and intercept throughout the experiment day. The CV of the slope, however, showed tendencies toward sex-based difference (*p* < 0.1) from days 7 to 12.

**Fig. 5:**
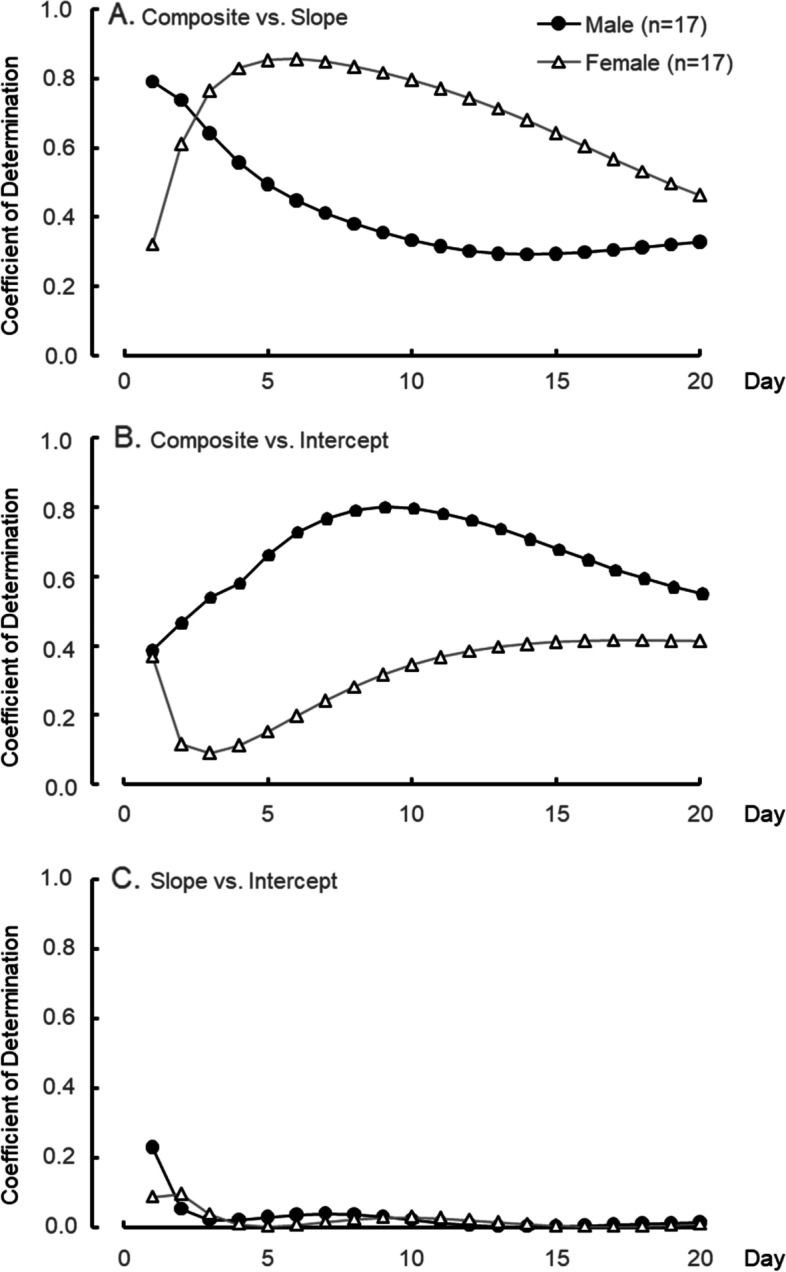
Coefficient of determination (CD) between the components for 20 days. CD between composite of main- and sub-process and main-process “slope” (upper), CD between the composite and sub-process “intercept” (middle), and CD between the slope and the intercept (lower). Black circles are male and white triangles are female

Figure 5 shows the CD among the indices over the experiment days. Although male subjects’ CD between the composite and the slope (CD–CP) got lower over the day, female subjects’ CD–CP increased until the fifth day and then became lower to the last day. CD between the composite and the intercept (CD–CI) in male subjects was higher than that in female subjects’ CD–CI throughout the experimental days. CD between the slope and the intercept (CD–SI) was almost 0 on all the days.

**Fig. 6:**
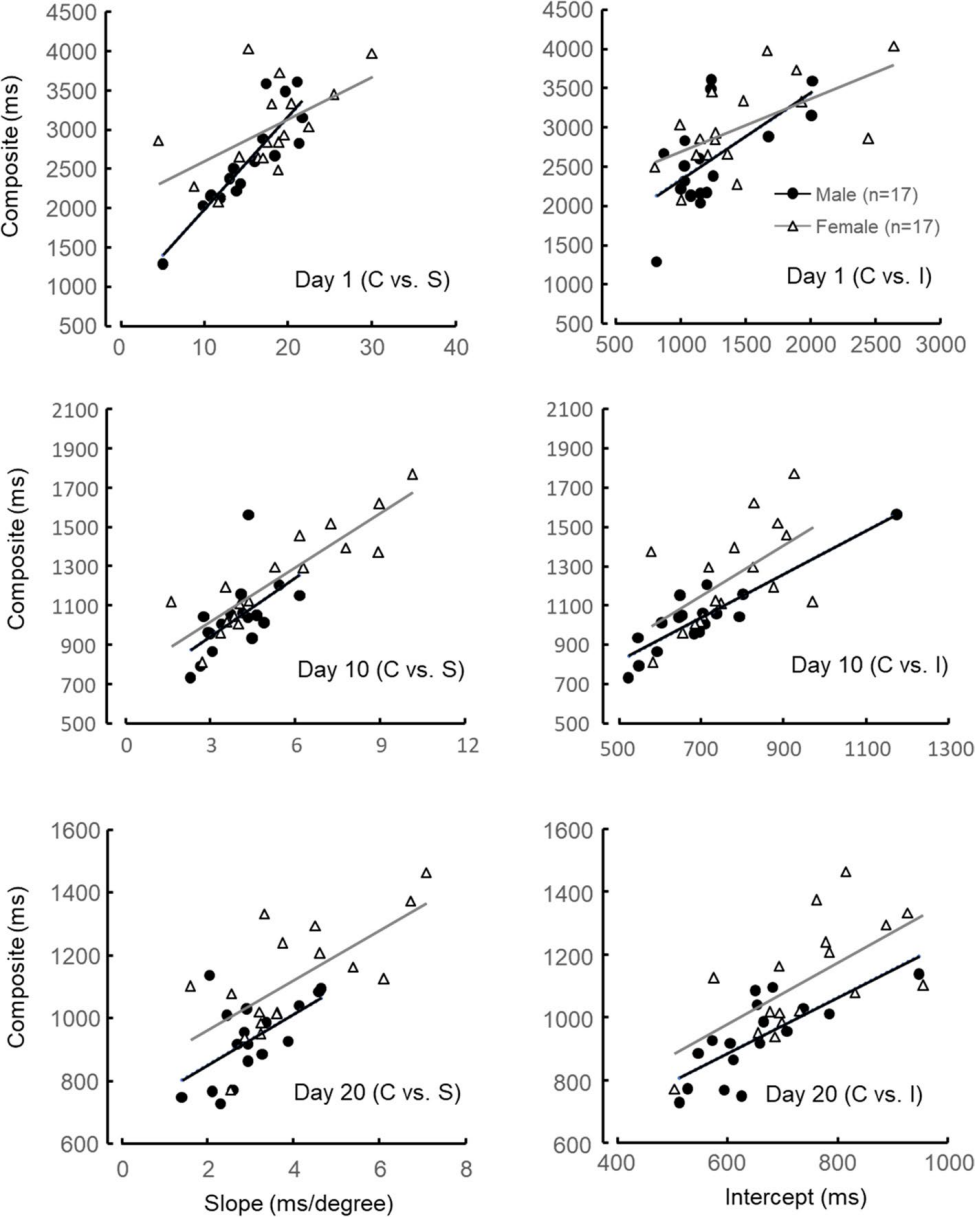
Scatter plots on day 1 (upper), day 10 (middle), and day 20 (lower). On the right-hand side are scatter plots between composite of main- and sub-processed and sub-process “intercept,” the left-hand side are those between the composite and main-process “slope”

The scatter plots between the composite and the slope and between the composite and the intercept on days 1, 10, and 20 are shown in Fig. 6. Some female subjects showed larger values of the slope than male subjects did on days 10 and 20.

## Discussion

Mean values of the three indexes on both sexes significantly decreased as the days went by, meaning that the subjects improved mental rotation and other sub-processes for solving the MRT through repetition. Although ANOVA showed significant main effects of sex on the mean values of the composite and slope, there was no significant interaction between sex and day. This means that the sex-based differences remained on the mental rotation during the experimental period. The CV of the slope for female subjects became larger than the CV for male subjects from days 7 to 12, indicating a larger interindividual difference in mental rotation in female subjects. Some females showed larger slope values than male subjects did on day 10 (left-hand side middle in Fig. 6). Female subjects who could hardly ameliorate the significant difference between the sexes in the mental rotation might have caused it.

The mean value of the intercept and the number of trials with incorrect responses reflected a tendency for sex exerting the main effect. The intercept involves sub-processes other than mental rotation, such as motor response. These findings indicated that male subjects, when compared with female subjects, tended to respond quickly but incorrectly. It might be caused by sex-based differences in risk-taking behavior. Some studies have argued that males are more likely to take risks such as financial risk [23] and physical risk [24] than females are. However, an earlier study [9] suggests that sex-based differences in the performance of the SM-MRT involve not only mental rotation but also other sub-processes (e.g., comparing the terminal arm direction of the figures or counting blocks of the figures). In an earlier study [9], various kinds of figures were used: mirror-image figures and structurally different figures. Our present study used only mirror figures structurally similar to the figures in invalid trials. Sex-based differences in non-rotation processes might be smaller in the SM-MRT configured with only mirror-image figures.

The CD–SIs on both male and female subjects were at a low level (almost 0) through the experimental period. This means that the slope index’s sub-process is completely different from that of the intercept and those in our previous studies [6, 12]. On the first day, although CD–CIs of both sexes were almost at the same level (0.4), the CD–CSs of male subjects were at a higher level (0.8) than those of female subjects (0.4). From these CDs, male subjects might be inferred to employ mainly mental rotation for solving the SM-MRT, while female subjects could be said to probably do both mental rotation and other sub-processes. Although people who have had experience with spatial experiments, such as special games, sports, and art, since junior high school students were recruited as subjects in this study, the frequency of their spatial activities before the experiment was not checked. Some studies have indicated that boys and young male subjects engage in more physical activity (including sports) and video games than girls and young female subjects [25–27]. Male subjects could recruit the rotation strategy on the first day because of their habitual spatial activities. After the second day, although the CD–CI of male subjects maintained a high level (above 0.6), the CD–CS was getting lower (below 0.5). Male subjects might employ other sub-processes as well as mental rotation. In an earlier study [9], when the subjects were told about the strategy of non-mental rotation in solving the SM-MRT, they could apply it immediately. Some male subjects in this study could learn a strategy of non-mental rotation after the second day. However, the CD–CS in female subjects was higher until the fifth day. This means that female subjects could solve the MRT mainly by mental rotation. Mathematics test scores have been reported to be related to the Vandenberg-MRT score [28]. Female subjects majoring in science and engineering outperformed the Vandenberg-MRT compared with those majoring in social sciences [15]. Since all of the female subjects in this study had majored in design science, it may have been easier for some of them to get a strategy of mental rotation. On the last day, CD–CSs and CD–CIs in both sexes converged to 0.4–0.6, meaning that most of them could gain both mental rotation and other sub-process and then employ them for solving the SM-MRT. This suggests that female subjects could have this ability to solve SM-MRT just as well as male subjects would if they continued the training.

## Conclusion

Although there were sex-based differences in strategies for solving the SM-MRT on the first day, both sexes might have employed both mental rotation and other sub-process after 20 days of repetition of the task. Present findings suggest that the performances obtained from the initial few trials of SM-MRT indicate temporary abilities that depend on the experiences of each individual at the time. However, the mean value of the slope (rotation speed) showed sex-based differences throughout the experimental period. The sex difference was caused by some female subjects who showed lower values of the slope. The learning rate of mental rotation may be one of the inherent spatial abilities. Since some studies suggest that MRT scores relate to academic major and/or spatial experience, further studies should examine the learning rate in other populations.

## Data Availability

The individual raw data will not be available because of the ethics policy.
